# A Novel Extracellular Matrix Gene-Based Prognostic Model to Predict Overall Survive in Patients With Glioblastoma

**DOI:** 10.3389/fgene.2022.851427

**Published:** 2022-06-17

**Authors:** Chen Qian, Wu Xiufu, Tang Jianxun, Chen Zihao, Shi Wenjie, Tang Jingfeng, Ulf D. Kahlert, Du Renfei

**Affiliations:** ^1^ Department of Cerebrovascular Diseases, The Second Affiliated Hospital of Guilin Medical University, Guilin, China; ^2^ University Hospital for Gynecology, Pius-Hospital, University Medicine Oldenburg, Oldenburg, Germany; ^3^ Molecular and Experimental Surgery, Medical Faculty University Hospital Magdeburg, University Clinic for General-, Visceral-, Vascular- and Trans-Plantation Surgery, Otto-von Guericke University, Magdeburg, Germany; ^4^ Clinic of Neurosurgery, Medical Faculty, Heinrich-Heine University Düsseldorf, Düsseldorf, Germany; ^5^ Chifeng Municipal Hospital, Inner Mongolia, Chifeng, China

**Keywords:** Glioblastoma, machine learning, ECM, survival prediction, web server

## Abstract

**Background:** Glioblastoma (GBM), one of the most prevalent brain tumor types, is correlated with an extremely poor prognosis. The extracellular matrix (ECM) genes could activate many crucial pathways that facilitate tumor development. This study aims to provide online models to predict GBM survival by ECM genes.

**Methods:** The associations of ECM genes with the prognosis of GBM were analyzed, and the significant prognosis-related genes were used to develop the ECM index in the CGGA dataset. Furthermore, the ECM index was then validated on three datasets, namely, GSE16011, TCGA-GBM, and GSE83300. The prognosis difference, differentially expressed genes, and potential drugs were obtained. Multiple machine learning methods were selected to construct the model to predict the survival status of GBM patients at 6, 12, 18, 24, 30, and 36 months after diagnosis.

**Results:** Five ECM gene signatures (AEBP1, F3, FLNC, IGFBP2, and LDHA) were recognized to be associated with the prognosis. GBM patients were divided into high– and low–ECM index groups with significantly different overall survival rates in four datasets. High–ECM index patients exhibited a worse prognosis than low–ECM index patients. Four small molecules (podophyllotoxin, lasalocid, MG-262, and nystatin) that might reduce GBM development were predicted by the Cmap dataset. In the independent dataset (GSE83300), the maximum values of prediction accuracy at 6, 12, 18, 24, 30, and 36 months were 0.878, 0.769, 0.748, 0.720, 0.705, and 0.868, respectively. These machine learning models were provided on a publicly accessible, open-source website (https://ospg.shinyapps.io/OSPG/).

**Conclusion:** In summary, our findings indicated that ECM genes were prognostic indicators for patient survival. This study provided an online server for the prediction of survival curves of GBM patients.

## Introduction

Glioblastoma (GBM), grade IV glioma, accounts for around 40–50 percent of brain tumors in America (Ostrom et al., 2017). There is an extremely unfavorable prognosis for GBM. Only about 5% of GBM patients will survive more than 5 years after diagnosis (Delgado-Lopez and Corrales-Garcia, 2016; Monteiro et al., 2017). Radiation exposure, particularly during childhood, is a risk factor for developing GBM (Roviello et al., 2013). The combination of surgery and temozolomide (TMZ) has been shown to prolong survival times. However, an increasing number of patients with GBM will develop resistance to TMZ after treatment (Dai et al., 2017). Consequently, it is critical to find novel biomarkers capable of accurately predicting prognosis and select appropriate individualized treatment strategies for patients with GBM.

The extracellular matrix (ECM) is mainly produced by fibroblasts and can be classified into two groups: fibrous proteins (collagen and fibronectin) and glycosaminoglycan (hyaluronic acid and chondroitin sulfate) (Brassart-Pasco et al., 2020; Huang et al., 2021). As a result of the strong crosslinking between these molecules, the ECM forms a dense mesh structure within the tissues. The interaction between cancer cells and ECM molecules activates many crucial pathways that facilitate the development of cancer. The presence of ECM components may represent a measure of tumor activity and invasiveness and could be used as a biomarker of disease (Brassart-Pasco et al., 2020). Therefore, a comprehensive understanding of ECM dysregulation in the tumor microenvironment (TME) will help identify potential GBM treatments.

This study is aimed at examining the association of ECM genes with GBM survival and providing models to predict survival. A total of five ECM genes were selected using bioinformatics analysis to build an ECM index model. A high ECM index was found to be associated with poorer overall survival. Potential drugs were predicted to reverse the negative prognosis of high–ECM index patients. More importantly, machine learning models were constructed to predict the survival status of 6, 12, 18, 24, 30, and 36 months. A free and user-friendly web server based on these machine learning models was provided in this study.

## Methods and Materials

### Data Curation Process

The available transcriptome and clinical data of GBM patients from the Chinese Glioma Genome Atlas (CGGA), Gene Expression Omnibus (GEO), and The Cancer Genome Atlas (TCGA) were used as the datasets for finding prognostic biomarkers and constructing prediction models. The samples, whose histology grade was grade IV or diagnosed with GBM, were downloaded and kept for analysis. There were 237 GBM samples from the CGGA dataset (Zhao et al., 2021), 150 GBM samples from GSE16011 (Gravendeel et al., 2009), and 159 GBM samples from the TCGA-GBM project. Another independent GBM dataset (GSE83300) that contained 50 GBM patients was used as the validation dataset for evaluating the performance of prediction models (Feng et al., 2017). There was a median survival time of 12, 8.7, 9, and 16.8 months in the CGGA, GSE16011, TCGA-GBM, and GSE83300 datasets, respectively. All the accession IDs of used samples from the four datasets are displayed in the Supplementary Table S1.

### Construction and Validation of the ECM Index

The expression values of genes in each dataset were normalized by a min–max normalization method using the following formula: z_i_ = (x_i_–min(x))/(max(x)—min(x)). Here, z_i_ is the normalized value of the gene expression, x_i_ is the expression value before normalization, min(x) is the minimum value of the gene expression in the dataset, and max(x) is the maximum value of the gene expression in the dataset. After normalization, the gene values range from 0 to 1. We used a univariate Cox regression analysis in CGGA, GSE16011, and TCGA-GBM to examine the links between ECM gene (1,936 unique ECM-related genes from 47 ECM gene sets) expression and overall survival (OS). The prognostic ECM genes were defined as the genes with *p*-value<0.05 in the univariate Cox regression analysis. The prognostic ECM genes were overlapped among the three datasets. Then, CGGA was set as the training dataset, GSE16011 and TCGA-GBM were set as the internal testing dataset, and GSE83300 was set as the independent validation dataset. The LASSO method from the “glmnet” package was adopted in CGGA to select the prognostic genes with a high importance value (Friedman et al., 2010). The ECM index was calculated by applying a multivariate Cox regression analysis to calculate the regression coefficient of genes.

The ECM index was calculated using the following formula: ECM index = *β*1* Gene_1 +…+ βn*Gene_n. In the formula, *β* is the regression coefficient and Gene_n is the expression value of genes. In the training, testing, and validation datasets, the patients were classified as high or low index based on the median value of the ECM index. Subsequently, we calculated the log-rank test and plotted the Kaplan–Meier curve to evaluate the difference in OS between the high– and low–ECM index groups. Then, the OS prediction ability of the ECM index was assessed by the area under the curve (AUC) at 12, 18, 24, and 36 months.

### Comparison of Expression Values Among Normal Brain, Low-Grade Glioma, and GBM Samples

The expression values of the selected genes were compared between the GBM and normal brain samples from the GEPIA web server (Tang et al., 2017). The GEPIA web server contains 163 GBM from the TCGA-GBM project and 207 normal brain samples from the GTEx project. For comparison of LGG and GBM, we downloaded and used the expression profiles of 159 GBM samples and 500 LGG samples from the TCGA-GBM and TCGA-LGG projects, respectively.

### Correlation Analysis of the ECM Index With Immune and Stromal Cell Populations

The MCP-counter which is capable of estimating the absolute abundance of eight immune and two stromal cell populations in the tumor samples by the expression data was used in this study (Becht et al., 2016). The differences in the immune and stromal cell populations were compared between the high– and low–ECM index groups using t-test analysis.

### Differential Gene Expression Analysis and the Identification of Potential Drugs

In CGGA, GSE16011, and TCGA-GBM, the differences in the expression levels of protein coding genes between the high–ECM index group samples and low–ECM index group samples for each ECM gene were analyzed using the linear models from the “limma” R package (Ritchie et al., 2015). We defined the cutoff criteria as *p*-value<0.05 and log2 fold change>0.5 to filter the statistically significant differentially expressed genes (DEGs) in each dataset. The DEGs were overlapped among the three datasets to obtain the robust DEGs. By using the Connectivity Map (CMap) database, we were able to examine the potential drugs with a close correlation to the diseases. The robust DEGs between the low– and high–ECM index groups were submitted to the Cmap database. The connectivity value from the Cmap represents the ability of the drug to reduce ECM formation, and the optimal connectivity value should be −1.

### Construction of Machine Learning Models and Web-Based Survival Rate Calculator

Machine learning methods were used in the construction of models to predict the survival rates of GBM patients at 6, 12, 18, 24, 30, and 36 months after diagnosis. Four machine learning algorithms, including support vector machine (SVM), random forest (RF), generalized linear models (GLM), and artificial neural network (ANN) from the caret R package were considered. To identify the best parameters for model creation, five-fold cross-validation and grid search were utilized. Data merging was performed on the CGGA, GSE16011, and TCGA-GBM datasets, and the samples were separated into the training dataset (60%) and the testing dataset (40%). GSE83300 was set as the independent validation dataset. After setting the best parameters, the models were trained on the training dataset and evaluated on the testing dataset and an independent validation dataset. The receiver operating characteristic (ROC) curve, a plot of sensitivity versus (1–specificity), was used to estimate the performance of models. We used the R package “shiny” to develop a web-based survival rate calculator and survival curve predictor using these machine learning models.

## Results

### Determination of Prognostic ECM Genes

**FIGURE 1:**
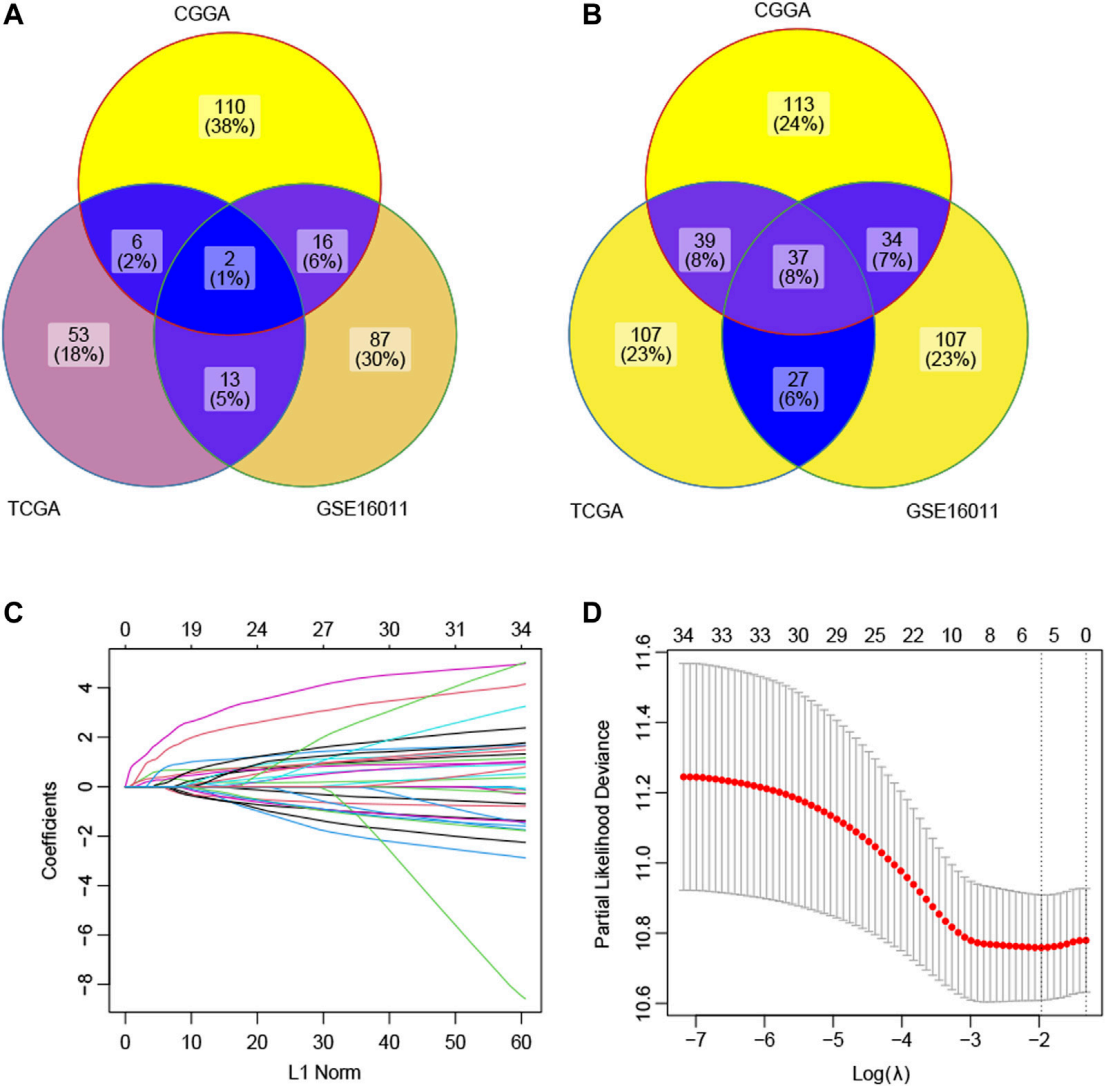
Selection of the prognostic ECM genes by the lasso method. **(A)** Intersection of the candidate protective genes. **(B)** Intersection of the candidate risky genes. **(C)** Lasso coefficient profiles of 39 genes were generated by comparing with the λ. Following the change of λ, the coefficients of the unimportant genes against the L1-norm (regularization term) in the model will be reduced to zero. The number of curves is the number of genes with non-zero coefficients at the current λ. **(D)** This plot is to show the cross-validation curve along with upper and lower standard deviation. The left vertical line indicates the gene number when the value of λ gives a minimal mean squared error (MSE). The right vertical line indicates the gene number when the cross-validated error is within one standard error of the minimum. Based on the first vertical line, the model included five genes with a non-zero prediction value.

In this study, 47 ECM gene sets containing 1,936 unique ECM-related genes were obtained (Supplementary Table S2). Among these 1,936 ECM-related genes, by the univariate Cox regression analyses, 134 genes were found to be positively correlated with prognosis in CGGA, 118 genes were found to be positively correlated with prognosis in GSE16011, and 74 genes were found to be positively correlated with prognosis in TCGA-GBM. These genes were identified as protective genes. Similarly, 223 genes were negatively correlated with prognosis in the CGGA dataset, 205 genes were negatively correlated with prognosis in the GSE16011 dataset, and 211 genes were negatively correlated with prognosis in the TCGA-GBM dataset. These genes were selected as risky genes. The intersection of the results showed that there were two common protective genes and 37 common risky genes (Figures 1A,B).

### Construction of the ECM Index

**FIGURE 2:**
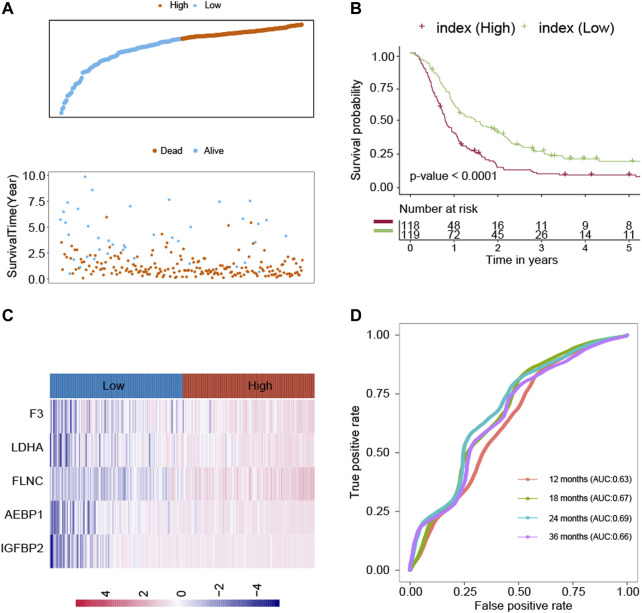
Construction of the ECM index in the CGGA dataset. **(A)** ECM index distribution and overall survival status of the GBM patients. **(B)** Kaplan–Meier survival curves of the high– and low–ECM index groups. **(C)** Gene expression profiles of ECM genes in the high– and low–ECM index groups. **(D)** AUC values for 12-, 18-, 24-, and 36-month overall survival predictions for the ECM index. Extracellular Matrix (ECM); Glioblastomas (GBM); area under the curve (AUC).

The LASSO technique was used on these 39 prognostic ECM genes in the CGGA, and the results indicated that AEBP1, F3, FLNC, IGFBP2, and LDHA were the best combination of genes to construct the model (Figures 1C,D). A multivariate Cox regression analysis calculated the regression coefficient. The ECM index was obtained by the following formula: [1.0391×AEBP1] + [0.6346×F3] + [0.5396×FLNC] + [2.1276×IGFBP2] + [2.7396×LDHA]. All the five selected genes (AEBP1, F3, FLNC, IGFBP2, and LDHA) were the risky genes since their regression coefficients were positive. The median value of the ECM index was selected to divide the patients into high– and–low ECM index groups. In Figure 2A, the ECM index distribution and overall survival data in the CGGA dataset were displayed and ranked according to the index value. The survival analysis indicated that the low–ECM index group had an apparent better prognosis than the high–ECM index group (*p*-value<0.05; Figure 2B). In Figure 2C, gene expression profiles of the high and low index groups were illustrated. The AUC values for 12-, 18-, 24-, and 36-month OS predictions were 0.63, 0.67, 0.69, and 0.66, respectively, which indicate its high ability to predict the prognosis of GBM (Figure 2D).

### Survival Analysis

In CGGA, GSE16011, and TCGA-GBM, the GBM patients were classified into two groups by the median gene expression of the five selected genes (AEBP1, F3, FLNC, IGFBP2, and LDHA). Then, survival analysis was used to assess the relationship between the gene expression value and OS. There were significant negative correlations between the expression levels of AEBP1, F3, FLNC, and LDHA in CGGA (*p*-value<0.05; Supplementary Figure S1). The findings were then validated in the GSE16011 and TCGA-GBM datasets. In GSE16011, the gene expression levels of AEBP1, FLNC, and LDHA were negatively correlated with OS (Supplementary Figure S2). In TCGA-GBM, there were significant negative correlations between the expression levels of AEBP1 and FLNC with OS (*p*-value<0.05; Supplementary Figure S3).

The expression values of AEBP1, F3, FLNC, IGFBP2, and LDHA were compared among normal brain tissue, low-grade gliomas (LGG), and GBM. The results from the GEPIA dataset showed that the expression values of these genes were higher in the GBM samples than in the normal brain samples (*p*-value<0.0001; Supplementary Figure S4). The expression values of these genes were also significantly higher in the GBM samples than in the LGG samples (*p*-value<0.0001; Supplementary Figure S5).

### Evaluation of the ECM Index Model

**FIGURE 3:**
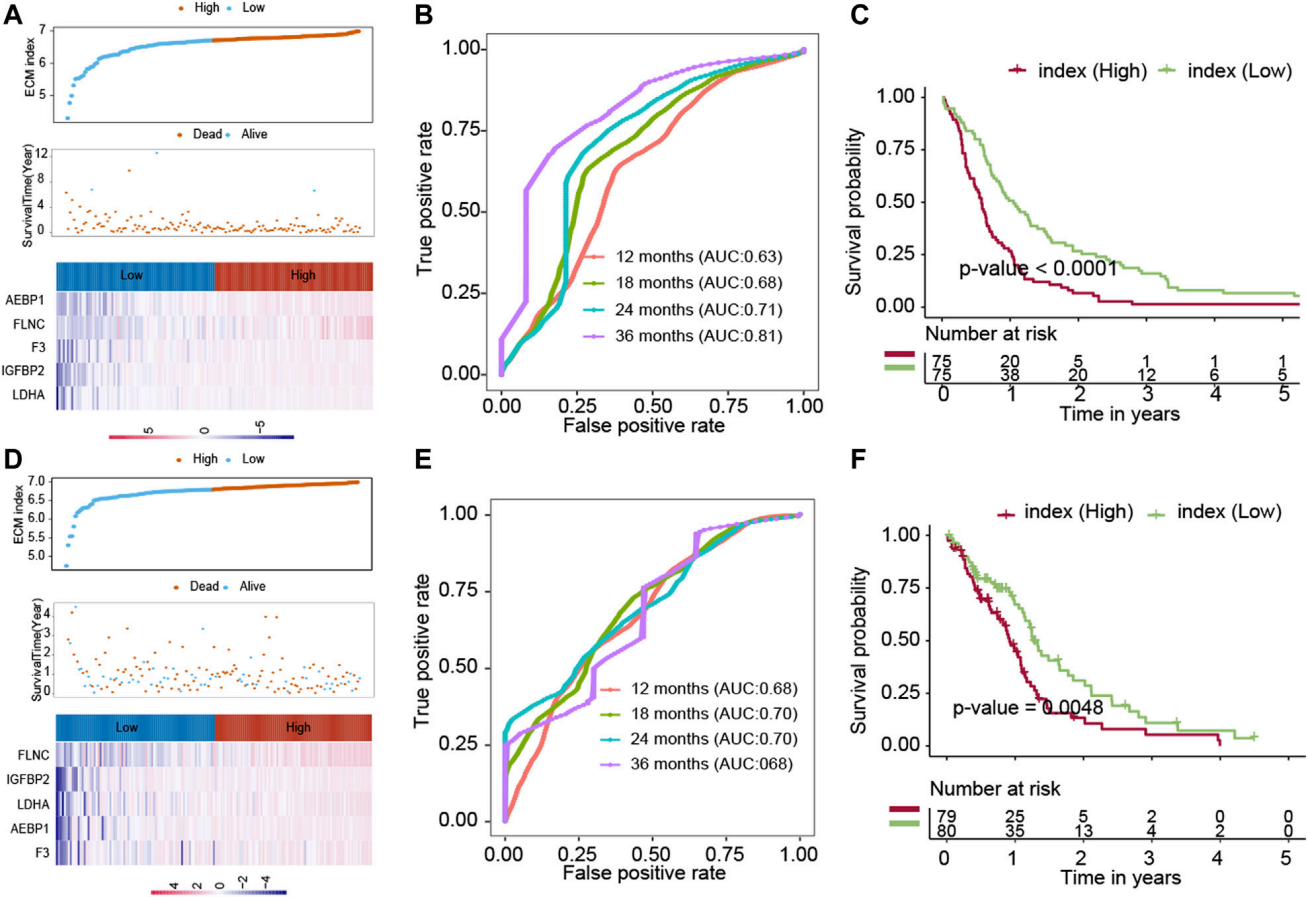
Evaluation of the ECM index in the internal validation datasets (GSE16011 and TCGA-GBM). The ECM indexes of all patients in the GSE16011 dataset **(A–C)** and the TCGA-GBM **(D–F)** dataset were calculated by the same formula and were divided into a high- and a low-index group.

To further evaluate the robustness of the ECM index, the ECM index of the samples in the GSE16011 and TCGA-GBM datasets was obtained using the same formula. The median value of the ECM index was also used to stratify the samples into the high– and low–ECM index groups. The ECM index distribution, overall survival, and ECM gene expression profiles in these two validation sets are plotted in Figure 3A, D. The ROC analysis results in the GSE16011 and TCGA-GBM datasets suggested that the ECM index had a powerful ability to predict GBM survival (Figures 3B,E). Compared to the high–ECM index patients, the low–ECM index patients had better overall survival prognosis (GSE16011: *p*-value<0.0001; TCGA-GBM: *p*-value = 0.0048; Figures 3C,F).

**FIGURE 4:**
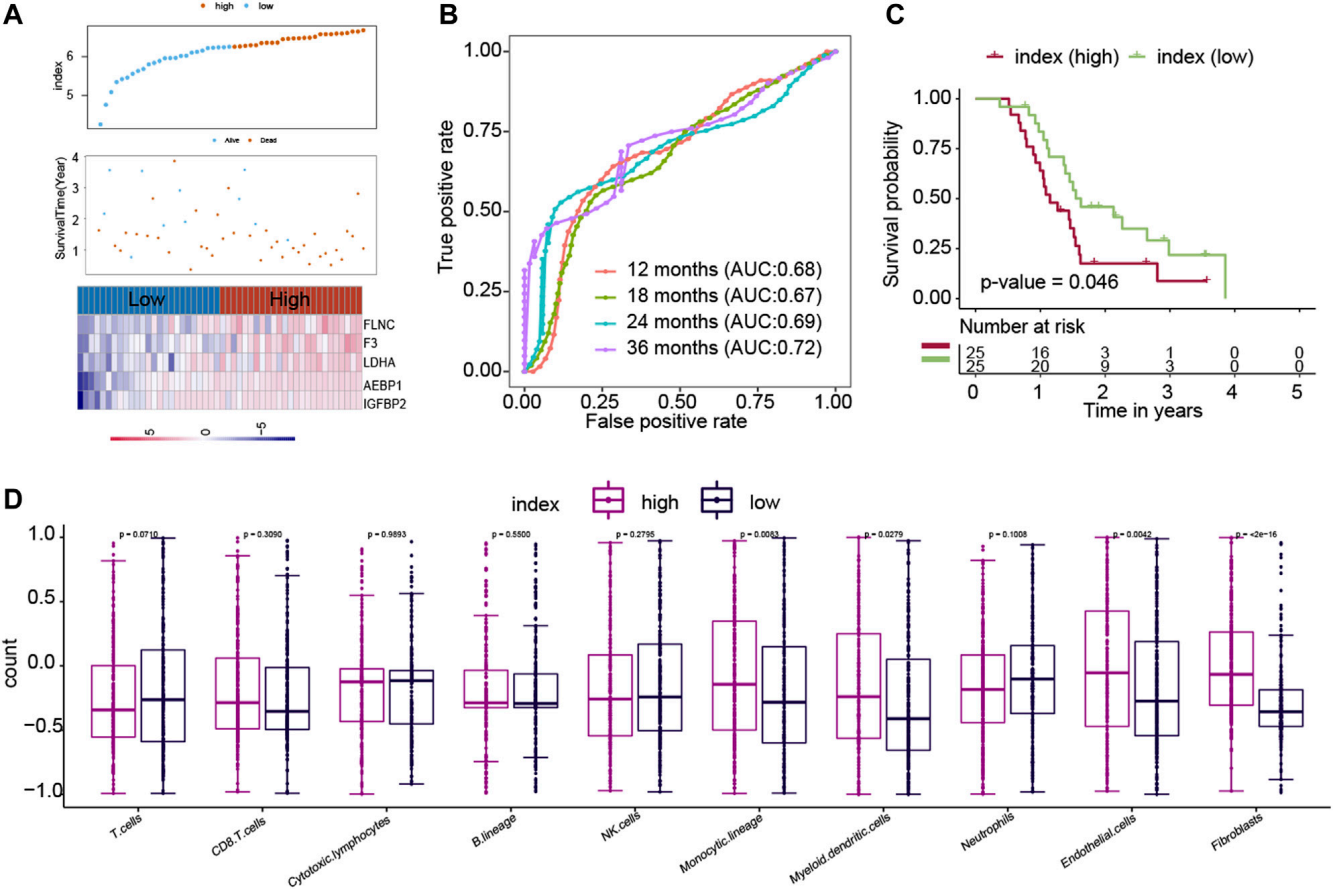
Evaluation of the ECM index in the independent dataset (GSE83300). The ECM index of all patients in the GSE83300 dataset **(A–C)** was calculated by the same formula and was divided into a high- and a low-index group. **(D)** Comparison of immune cells and stromal cells between than the low– and high–ECM index patients.

Another independent dataset (GSE83300), which contains 50 GBM patients, was used to validate the results. The ECM index distribution, overall survival, and ECM gene expression profiles in GSE83300 are plotted in Figure 4A. The ROC analysis results suggested that the ECM index had a powerful ability to predict GBM survival (Figure 4B). Compared to high–ECM index patients, the low–ECM index patients had better overall survival prognosis (*p*-value = 0.046; Figure 4C), which is in concordance with the results from the CGGA, GSE16011, and TCGA-GBM datasets.

### Endothelial Cells and Fibroblasts Were Higher in the High–ECM Index Group Samples

We calculated the association between the ECM index and population abundances of the immune and stromal cells in the combined CGGA, GSE16011, and TCGA-GBM datasets. As shown in Figure 4D, the high index group samples were found to comprise higher endothelial cells and fibroblast.

### Identifying DEGs and Screening Potential Drugs

Data profiles from CGGA, GSE16011, and TCGA-GBM were used to find the DEGs between the high– and low–ECM index groups. DEGs calculated from the three datasets were overlapped to obtain the robust DEGs, and a total of 286 robust DEGs were identified. To predict the potential drugs for GBM, CMap was implemented on the robust DEGs between the low– and high–ECM index groups. The 20 drugs with high and significant associations with robust DEGs are listed in Supplementary Table S3. Among these drugs, podophyllotoxin, lasalocid, MG-262, and nystatin revealed higher negative correlations and the potential to reverse the high ECM index tumor status.

### Development of Machine Learning Models to Predict GBM Patient Survival

**FIGURE 5:**
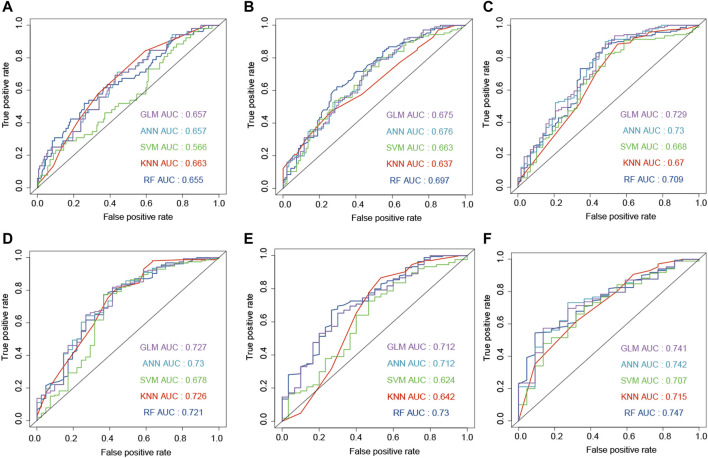
Development of the machine learning models to predict the survival status at 6 **(A)**, 12 **(B)**, 18 **(C)**, 24 **(D)**, 30 **(E)**, and 36 **(F)** months based on gender, age, and ECM genes (AEBP1, F3, FLNC, IGFBP2, and LDHA). The models were trained on the training dataset, and these AUC results were calculated on the testing dataset.

**FIGURE 6:**
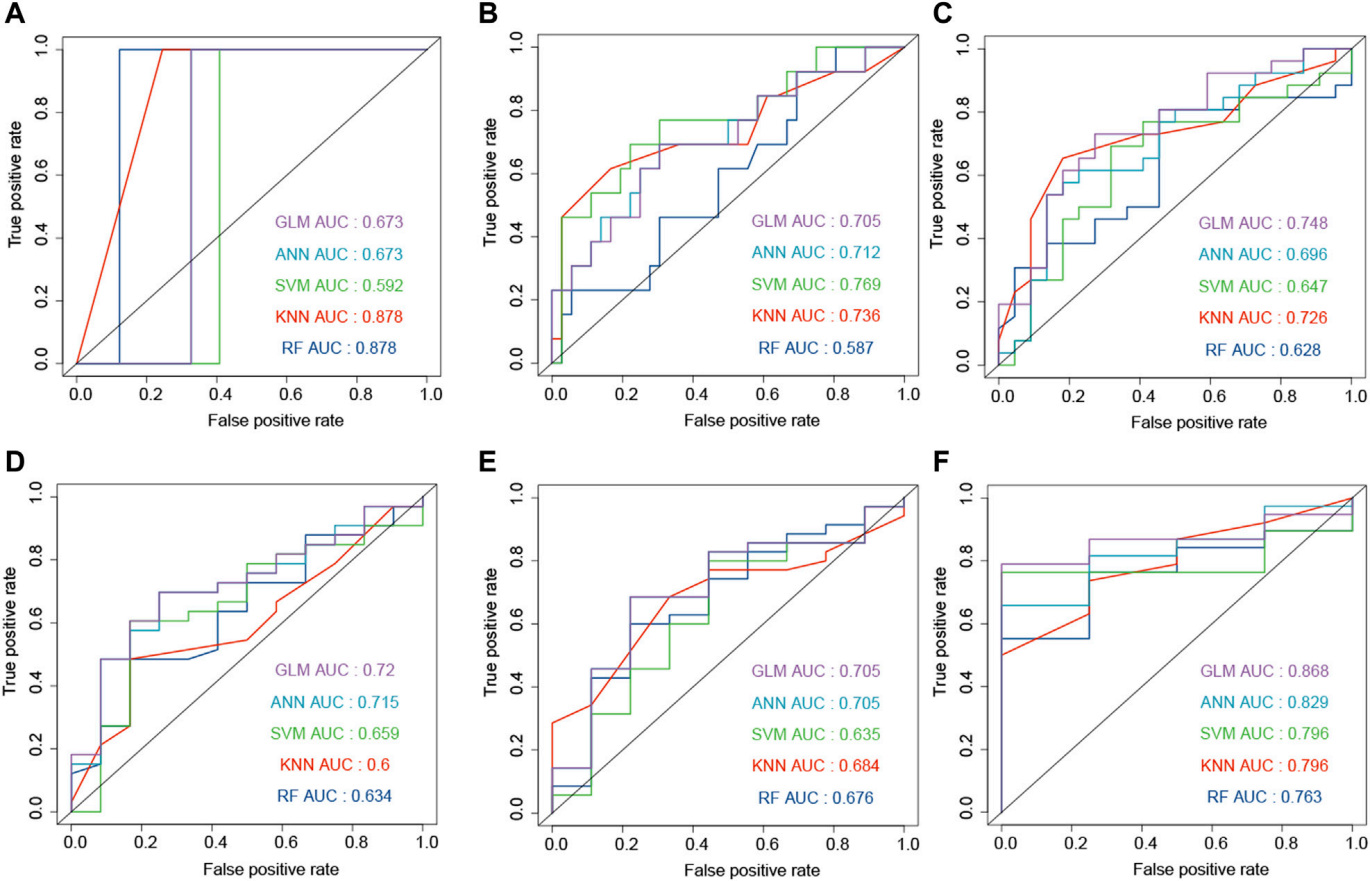
Development and evaluation of the machine learning models to predict the survival status at 6 **(A)**, 12 **(B)**, 18 **(C)**, 24 **(D)**, 30 **(E)**, and 36 **(F)** months based on gender, age, and ECM genes (AEBP1, F3, FLNC, IGFBP2, and LDHA). These AUC results were calculated on the independent dataset (GSE83300).

The combination of genes, age, and gender was used to predict the survival status of GBM patients. The survival status of 6, 12, 18, 24, and 30 months was predicted by the machine learning methods. First, CGGA, GSE16011, and TCGA-GBM were combined into one dataset. Then, the combined dataset was randomly divided into training (60%) and testing (40%) datasets. The commonly used machine learning classifiers, including GLM, ANN, SVM, KNN, and RF, were constructed to predict the survival status of patients at 6, 12, 18, 24, 30, and 36 months after the treatment, respectively. After setting the best parameter *via* a five-fold cross-validation strategy, we calculated the AUC value in the testing dataset to characterize the ability of the model to distinguish between the dead and alive. KNN, RF, ANN, ANN, RF, and RF demonstrated the highest AUC values in predicting the survival status at 6, 12, 18, 24, 30, and 36 months (Figures 5A–E, F), respectively. Then, these constructed machine learning models were also validated by an independent dataset (GSE83300). RF, SVM, GLM, GLM, ANN, and GLM demonstrated the highest AUC values in predicting the survival status at 6, 12, 18, 24, 30, and 36 months (Figures 6A–F), respectively.

### A Web-Based Interactive Tool for Predicting Survival Probability and Plotting the Survival Curve

**FIGURE 7:**
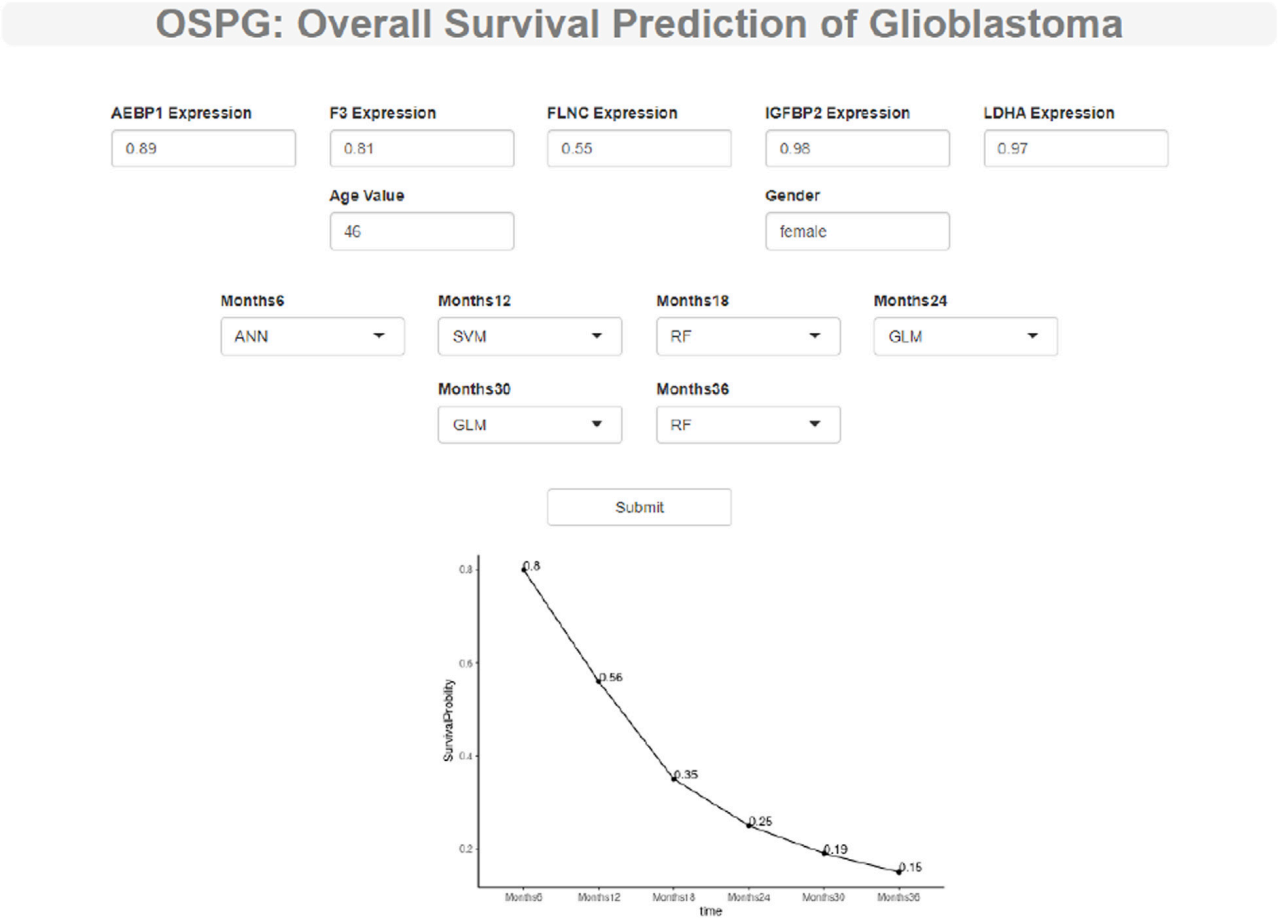
OSPG: a web tool to explore and predict the overall survival status of the glioblastoma patients. OSPG can be used in four steps: (1) *via* the website https://ospg.shinyapps.io/OSPG/, (2) inputting the values of five genes including AEBP1, F3, FLNC, IGFBP2, and LDHA (gene expression values range 0 to 1), (3) inputting the values of age and gender (male or female) including AEBP1, F3, FLNC, IGFBP2, and LDHA (gene expression values range 0 to 1), (4) selecting the machine learning models for each time point (6, 12, 18, 24, 30, 36 months), and (5) clicking the “submit” button.

To make these constructed machine learning models accessible to GBM researchers, we developed a web tool to predict the survival probability of GBM patients called OSPG. OSPG can be used in five steps 1) visiting the website https://ospg.shinyapps.io/OSPG/;(2) inputting the values of five genes, including AEBP1, F3, FLNC, IGFBP2, and LDHA (gene expression values range 0–1); 3) inputting the values of age and gender (male or female); 4) selecting the machine learning models for each time point (6, 12, 18, 24, 30, and 36 months), and 5) clicking the “submit” button. An example is illustrated in Figure 7.

## Discussion

The prognosis of GBM is very poor; only about 5 percent GBM patients could survive more than 5 years (Delgado-Lopez and Corrales-Garcia, 2016). Currently, therapeutic options for GBM include surgery alone or adjuvant radio/chemotherapy in combination with surgical resection (Chen et al., 2020). Surgical resection is ineffective because cancer cells may have infiltrated the surrounding tissues or metastasized. In addition, it is still debatable whether systemic adjuvant medication can be administered following surgery due to the possibility of adverse effects (Abdul et al., 2018). As a result, it is critical to identify possible biomarkers for predicting GBM prognosis. The key findings of this study are as follows: 1). we identified five ECM genes associated with prognosis, and these five genes were used to calculate the ECM index. 2). We constructed the machine learning models to predict the survival status of the GBM patients at 6, 12, 18, 24, 30, and 36 months after treatment. 3). We provided a web server to predict the survival curves of GBM patients by these machine learning models.

ECM is one of the major components of the TME, and its interaction with the tumor cells promotes tumor growth and migration (Klank et al., 2017). Thus, computing the ECM scores in diverse cancers is critical for comprehending and potentially addressing various tumors thoroughly. In this study, we identified five ECM genes that are associated with prognosis and used their expression values to calculate the ECM index. The high ECM index was correlated with a negative prognosis. The predicted drugs, including podophyllotoxin, lasalocid, MG-262, and nystatin, might contribute to reversing the oncogenic roles of the ECM in GBM tumor development. Podophyllotoxin, a natural product, and its derivatives have the potential to inhibit cell growth by tubulin polymerization (Ardalani et al., 2017). Semisynthetic derivatives of podophyllotoxin have been used as therapies for cancers including leukemia, lymphoma, and GBM (Ardalani et al., 2017; Chen et al., 2013). Lasalocid could work against tumor cells by cell cycle arrest (Kim et al., 2017). MG-262 is a potent proteasome inhibitor, and more studies are needed to clarify the effect of MG-262 on tumor growth, especially GBM. Nystatin is a cholesterol-sequestering antifungal drug and could further prolong animal survival and significantly suppress tumor growth (Chen et al., 2015).

AEBP1 has the potential to bind with collagen and then contribute to collagen polymerization, which is critical for several biological processes such as tissue repair and fibrosis (Blackburn et al., 2018). The high levels of AEBP1 were found in collagen-rich tissues, including the dermal layer of the skin, the medial layer of blood vessels, and the basement membrane. Recent research has established that AEBP1 plays a critical function in carcinogenesis and progression. For example, the expression value of AEBP1 is higher in the glioma cells, and inhibition of AEBP1 could induce apoptosis of GBM cell lines (Ladha et al., 2012). F3 is the gene for encoding tissue factor (TF), and the proinvasive activity of F3 is positively correlated with the ECM–receptor interactions and the invasiveness of GBM cells (Unruh et al., 2019). FLNC is a cytoskeletal protein and could contribute to GBM metastasis by promoting ECM degradation (Kamil et al., 2019). The survival analysis results also found that the high FLNC expression was linked with a negative GBM prognosis. IGFBP2 is positively correlated with tumor grades and negatively associated with the prognosis of glioma patients (Lin et al., 2009). Similarly, LDHA expression levels and stage of the tumor are positively correlated (Di et al., 2018). The knockdown of LDHA decreased the cell growth by impairing cell cycle progression and triggering apoptosis in glioma cell lines. The cellular distribution of these selected genes was different. For AEBP1 (Layne et al., 2001), F3 (Indira et al., 2019), IGFBP2 (Gyuris et al., 2019), and LDHA (Lin et al., 2018), they are abundantly expressed in the extracellular components. However, the expression of FLNC results in cytoplasmic distribution mainly associated with actin fibers (Valdes-Mas et al., 2014).

Recently, a systematic review summarized the available models for the survival prediction of GBM (Tewarie et al., 2021). It is a challenging field since there are several problems: 1) GBM patients have a very poor prognosis. For example, in the current study, the median survival time of patients from CGGA, GSE16011, and TCGA-GBM datasets is 12, 8.7, and 9 months, respectively. The short survival time limits the ability of models to predict the prognosis. 2) Machine learning methods can only predict binary or continuous targets and are not accessible to the censored survival data, which contains survival time and survival status. In the article by Ishaan (Tewarie et al., 2021), according to the AUC (0.58–0.98), accuracy (0.69–0.98), and C-index (0.66–0.70), the prediction performance of 59 models differed greatly. For most of these models, it is hard to reach a strong sensitivity or specificity (AUC>0.8). Only seven of these models have been independently evaluated, and only three studies have converted their models into an online prediction tool. For example, a prediction model constructed by clinical information achieved a maximum C-index value of 0.66 (Gorlia et al., 2008). Another online survival probability and survival curve predictor for the patients with GBM that uses patient demographics and clinical characteristics was provided (C-index = 0.70) (Senders et al., 2020).

The advantages of this study were that it provided accurate models and constructed the web server. In the independent dataset, the machine learning models reached the AUC values of 0.878 and 0.868 in predicting the survival status at 6 and 36 months (Figure 6), respectively. In order to predict the survival curve of GBM, we constructed models to predict the survival probability of time points such as 6, 12, 18, 24, 30, and 36 months. Then, the survival curve was generated and plotted by the survival probability values.

Nevertheless, the present study has several limitations. First, although validation datasets were used in this study, the models need to be carefully assessed and validated by future prospective studies. For example, the survival status of GBM patients could be predicted by the machine learning models and expression profiles of these five genes. Then, the accuracy of our models could be validated by comparing the predicted and real survival status. Second, the roles of the selected genes in GBM also need to be tested by experiments.

## Conclusion

We identified the ECM genes with prognostic power and offered potential small-molecule drugs for the treatment of GBM. The online web server based on the five ECM genes could accurately predict the survival curves of GBM patients after the diagnosis, which could be particularly useful for tailoring clinical care to the needs of the individual GBM patient.

## Data Availability

The original contributions presented in the study are included in the article/Supplementary Material, further inquiries can be directed to the corresponding authors.
