# Supplementary material for: A Novel Extracellular Matrix Gene-Based Prognostic Model to Predict Overall Survive in Patients With Glioblastoma
Source: Front Genet. 2022 Jun 17;13:851427. doi: 10.3389/fgene.2022.851427 (PMC9247148; doi:10.3389/fgene.2022.851427)
Supplement: Supplementary file 1 [file Table1.docx]

| Dataset | ID |
| --- | --- |
| CGGA | "CGGA_1017" "CGGA_1036" "CGGA_1041" "CGGA_1075" "CGGA_1086" "CGGA_1103"  "CGGA_1106" "CGGA_1130" "CGGA_1134" "CGGA_1135" "CGGA_1138" "CGGA_1142"  "CGGA_1164" "CGGA_1172" "CGGA_120" "CGGA_1208" "CGGA_1236" "CGGA_1248"  "CGGA_1255" "CGGA_1256" "CGGA_1257" "CGGA_1260" "CGGA_1262" "CGGA_1282"  "CGGA_1326" "CGGA_1337" "CGGA_1353" "CGGA_1354" "CGGA_1365" "CGGA_1371"  "CGGA_1378" "CGGA_1380" "CGGA_1382" "CGGA_1387" "CGGA_139" "CGGA_1391"  "CGGA_1392" "CGGA_1402" "CGGA_1403" "CGGA_1410" "CGGA_1415" "CGGA_1418"  "CGGA_1419" "CGGA_1420" "CGGA_1422" "CGGA_1425" "CGGA_1426" "CGGA_1429"  "CGGA_1430" "CGGA_1433" "CGGA_1441" "CGGA_1444" "CGGA_1451" "CGGA_1452"  "CGGA_1457" "CGGA_1461" "CGGA_1462" "CGGA_1467" "CGGA_1472" "CGGA_1476"  "CGGA_1478" "CGGA_1480" "CGGA_1481" "CGGA_1486" "CGGA_1491" "CGGA_1492"  "CGGA_1494" "CGGA_1496" "CGGA_1500" "CGGA_1501" "CGGA_1503" "CGGA_1505"  "CGGA_1507" "CGGA_1520" "CGGA_1521" "CGGA_1529" "CGGA_1534" "CGGA_1535"  "CGGA_1537" "CGGA_1538" "CGGA_1539" "CGGA_1541" "CGGA_1542" "CGGA_1543"  "CGGA_1546" "CGGA_1548" "CGGA_1551" "CGGA_1558" "CGGA_1559" "CGGA_1560"  "CGGA_1564" "CGGA_1571" "CGGA_1572" "CGGA_1586" "CGGA_1595" "CGGA_1596"  "CGGA_1597" "CGGA_1601" "CGGA_1603" "CGGA_1604" "CGGA_1605" "CGGA_1611"  "CGGA_1612" "CGGA_1613" "CGGA_1615" "CGGA_1624" "CGGA_1626" "CGGA_1631"  "CGGA_1634" "CGGA_1635" "CGGA_1641" "CGGA_1644" "CGGA_1650" "CGGA_1656"  "CGGA_1658" "CGGA_1659" "CGGA_1663" "CGGA_1666" "CGGA_1678" "CGGA_1681"  "CGGA_1682" "CGGA_1687" "CGGA_1690" "CGGA_1694" "CGGA_1697" "CGGA_1698"  "CGGA_1699" "CGGA_1702" "CGGA_1706" "CGGA_1708" "CGGA_1709" "CGGA_1713"  "CGGA_1722" "CGGA_1727" "CGGA_1728" "CGGA_1729" "CGGA_1735" "CGGA_1736"  "CGGA_1740" "CGGA_1744" "CGGA_1749" "CGGA_1750" "CGGA_1758" "CGGA_1764"  "CGGA_1767" "CGGA_1769" "CGGA_1770" "CGGA_1773" "CGGA_1776" "CGGA_1780"  "CGGA_1785" "CGGA_1807" "CGGA_1811" "CGGA_1812" "CGGA_1814" "CGGA_1815"  "CGGA_1817" "CGGA_1819" "CGGA_1820" "CGGA_1826" "CGGA_1833" "CGGA_1840"  "CGGA_1857" "CGGA_1865" "CGGA_1866" "CGGA_1870" "CGGA_1886" "CGGA_1899"  "CGGA_1901" "CGGA_1908" "CGGA_1911" "CGGA_1912" "CGGA_1946" "CGGA_1953"  "CGGA_1955" "CGGA_1972" "CGGA_1976" "CGGA_1985" "CGGA_2003" "CGGA_2008"  "CGGA_2024" "CGGA_2039" "CGGA_2047" "CGGA_2053" "CGGA_2056" "CGGA_2062"  "CGGA_2075" "CGGA_2078" "CGGA_2082" "CGGA_2106" "CGGA_2115" "CGGA_487"  "CGGA_509" "CGGA_530" "CGGA_568" "CGGA_777" "CGGA_831" "CGGA_869"  "CGGA_P100" "CGGA_P102" "CGGA_P106" "CGGA_P109" "CGGA_P112" "CGGA_P116"  "CGGA_P136" "CGGA_P143" "CGGA_P15" "CGGA_P160" "CGGA_P164" "CGGA_P175"  "CGGA_P178" "CGGA_P180" "CGGA_P182" "CGGA_P199" "CGGA_P205" "CGGA_P22"  "CGGA_P25" "CGGA_P28" "CGGA_P280" "CGGA_P283" "CGGA_P295" "CGGA_P335"  "CGGA_P385" "CGGA_P401" "CGGA_P411" "CGGA_P415" "CGGA_P512" "CGGA_P585"  "CGGA_P596" "CGGA_P609" "CGGA_P610" "CGGA_P619" "CGGA_P625" "CGGA_P7"  "CGGA_P87" "CGGA_P89" "CGGA_P99" |
| GSE16011 | "GSM405213" "GSM405214" "GSM405215" "GSM405216" "GSM405217" "GSM405218"  "GSM405219" "GSM405220" "GSM405221" "GSM405222" "GSM405223" "GSM405224"  "GSM405228" "GSM405229" "GSM405230" "GSM405231" "GSM405232" "GSM405233"  "GSM405234" "GSM405235" "GSM405236" "GSM405237" "GSM405238" "GSM405239"  "GSM405240" "GSM405241" "GSM405242" "GSM405243" "GSM405244" "GSM405245"  "GSM405246" "GSM405247" "GSM405248" "GSM405249" "GSM405251" "GSM405252"  "GSM405253" "GSM405254" "GSM405255" "GSM405260" "GSM405262" "GSM405263"  "GSM405264" "GSM405266" "GSM405267" "GSM405268" "GSM405269" "GSM405270"  "GSM405271" "GSM405274" "GSM405275" "GSM405276" "GSM405278" "GSM405280"  "GSM405282" "GSM405290" "GSM405292" "GSM405293" "GSM405294" "GSM405296"  "GSM405297" "GSM405299" "GSM405301" "GSM405302" "GSM405303" "GSM405304"  "GSM405305" "GSM405307" "GSM405309" "GSM405312" "GSM405313" "GSM405314"  "GSM405315" "GSM405317" "GSM405320" "GSM405322" "GSM405323" "GSM405324"  "GSM405326" "GSM405328" "GSM405330" "GSM405337" "GSM405345" "GSM405349"  "GSM405350" "GSM405351" "GSM405352" "GSM405353" "GSM405356" "GSM405362"  "GSM405363" "GSM405365" "GSM405367" "GSM405368" "GSM405369" "GSM405370"  "GSM405371" "GSM405372" "GSM405373" "GSM405374" "GSM405375" "GSM405376"  "GSM405379" "GSM405391" "GSM405392" "GSM405393" "GSM405396" "GSM405397"  "GSM405405" "GSM405412" "GSM405415" "GSM405416" "GSM405417" "GSM405418"  "GSM405419" "GSM405422" "GSM405426" "GSM405427" "GSM405428" "GSM405430"  "GSM405431" "GSM405432" "GSM405434" "GSM405436" "GSM405438" "GSM405440"  "GSM405442" "GSM405443" "GSM405446" "GSM405447" "GSM405448" "GSM405452"  "GSM405453" "GSM405454" "GSM405455" "GSM405458" "GSM405459" "GSM405461"  "GSM405463" "GSM405464" "GSM405465" "GSM405466" "GSM405470" "GSM405471"  "GSM405472" "GSM405473" "GSM405474" "GSM405475" "GSM405477" "GSM405479" |
| TCGA | "TCGA.02.0047" "TCGA.02.0055" "TCGA.02.2483" "TCGA.02.2485" "TCGA.02.2486"  "TCGA.06.0125" "TCGA.06.0129" "TCGA.06.0130" "TCGA.06.0132" "TCGA.06.0138"  "TCGA.06.0139" "TCGA.06.0141" "TCGA.06.0152" "TCGA.06.0156" "TCGA.06.0157"  "TCGA.06.0158" "TCGA.06.0168" "TCGA.06.0171" "TCGA.06.0174" "TCGA.06.0178"  "TCGA.06.0184" "TCGA.06.0187" "TCGA.06.0190" "TCGA.06.0210" "TCGA.06.0211"  "TCGA.06.0219" "TCGA.06.0221" "TCGA.06.0238" "TCGA.06.0644" "TCGA.06.0645"  "TCGA.06.0646" "TCGA.06.0649" "TCGA.06.0686" "TCGA.06.0743" "TCGA.06.0744"  "TCGA.06.0745" "TCGA.06.0747" "TCGA.06.0749" "TCGA.06.0750" "TCGA.06.0878"  "TCGA.06.0882" "TCGA.06.1804" "TCGA.06.2557" "TCGA.06.2558" "TCGA.06.2559"  "TCGA.06.2561" "TCGA.06.2562" "TCGA.06.2563" "TCGA.06.2564" "TCGA.06.2565"  "TCGA.06.2567" "TCGA.06.2569" "TCGA.06.2570" "TCGA.06.5408" "TCGA.06.5410"  "TCGA.06.5411" "TCGA.06.5412" "TCGA.06.5413" "TCGA.06.5414" "TCGA.06.5416"  "TCGA.06.5417" "TCGA.06.5418" "TCGA.06.5856" "TCGA.06.5858" "TCGA.06.5859"  "TCGA.08.0386" "TCGA.12.0616" "TCGA.12.0618" "TCGA.12.0619" "TCGA.12.0821"  "TCGA.12.1597" "TCGA.12.3650" "TCGA.12.3652" "TCGA.12.3653" "TCGA.12.5295"  "TCGA.12.5299" "TCGA.14.0736" "TCGA.14.0781" "TCGA.14.0787" "TCGA.14.0789"  "TCGA.14.0790" "TCGA.14.0817" "TCGA.14.0871" "TCGA.14.1034" "TCGA.14.1402"  "TCGA.14.1823" "TCGA.14.1825" "TCGA.14.1829" "TCGA.14.2554" "TCGA.15.0742"  "TCGA.15.1444" "TCGA.16.0846" "TCGA.16.1045" "TCGA.19.0957" "TCGA.19.1389"  "TCGA.19.1390" "TCGA.19.1787" "TCGA.19.2619" "TCGA.19.2620" "TCGA.19.2624"  "TCGA.19.2625" "TCGA.19.2629" "TCGA.19.4065" "TCGA.19.5960" "TCGA.26.1442"  "TCGA.26.5132" "TCGA.26.5133" "TCGA.26.5134" "TCGA.26.5135" "TCGA.26.5136"  "TCGA.26.5139" "TCGA.27.1830" "TCGA.27.1831" "TCGA.27.1832" "TCGA.27.1834"  "TCGA.27.1835" "TCGA.27.1837" "TCGA.27.2519" "TCGA.27.2521" "TCGA.27.2523"  "TCGA.27.2524" "TCGA.27.2526" "TCGA.27.2528" "TCGA.28.1747" "TCGA.28.1753"  "TCGA.28.2509" "TCGA.28.2513" "TCGA.28.2514" "TCGA.28.5204" "TCGA.28.5207"  "TCGA.28.5208" "TCGA.28.5209" "TCGA.28.5213" "TCGA.28.5215" "TCGA.28.5216"  "TCGA.28.5218" "TCGA.28.5220" "TCGA.32.1970" "TCGA.32.1980" "TCGA.32.1982"  "TCGA.32.2615" "TCGA.32.2616" "TCGA.32.2632" "TCGA.32.2634" "TCGA.32.2638"  "TCGA.32.4213" "TCGA.32.5222" "TCGA.41.2571" "TCGA.41.2572" "TCGA.41.3915"  "TCGA.41.4097" "TCGA.41.5651" "TCGA.76.4925" "TCGA.76.4926" "TCGA.76.4927"  "TCGA.76.4928" "TCGA.76.4929" "TCGA.76.4931" "TCGA.76.4932" |
| GSE83300 | "GSM2198606" "GSM2198607" "GSM2198608" "GSM2198609" "GSM2198610" "GSM2198611"  "GSM2198612" "GSM2198613" "GSM2198614" "GSM2198615" "GSM2198616" "GSM2198617"  "GSM2198618" "GSM2198619" "GSM2198620" "GSM2198621" "GSM2198622" "GSM2198623"  "GSM2198624" "GSM2198625" "GSM2198626" "GSM2198627" "GSM2198628" "GSM2198629"  "GSM2198630" "GSM2198631" "GSM2198632" "GSM2198633" "GSM2198634" "GSM2198635"  "GSM2198636" "GSM2198637" "GSM2198638" "GSM2198639" "GSM2198640" "GSM2198641"  "GSM2198642" "GSM2198643" "GSM2198644" "GSM2198645" "GSM2198646" "GSM2198647"  "GSM2198648" "GSM2198649" "GSM2198650" "GSM2198651" "GSM2198652" "GSM2198653"  "GSM2198654" "GSM2198655" |

**Supplementary table 1.** The GBM samples used in this study.

| **Gene Set Name** | **Website** |
| --- | --- |
| NABA COLLAGENS | https://www.gsea-msigdb.org/gsea/msigdb/cards/NABA COLLAGENS |
| NABA ECM GLYCOPROTEINS | https://www.gsea-msigdb.org/gsea/msigdb/cards/NABA_ECM_GLYCOPROTEINS |
| NABA ECM REGULATORS | https://www.gsea-msigdb.org/gsea/msigdb/cards/NABA_ECM_REGULATORS |
| NABA ECM AFFILIATED | https://www.gsea-msigdb.org/gsea/msigdb/cards/NABA_ECM_AFFILIATED |
| NABA PROTEOGLYCANS | https://www.gsea-msigdb.org/gsea/msigdb/cards/NABA_PROTEOGLYCANS |
| NABA SECRETED FACTORS | https://www.gsea-msigdb.org/gsea/msigdb/cards/NABA_SECRETED_FACTORS |
| NABA CORE MATRISOME | https://www.gsea-msigdb.org/gsea/msigdb/cards/NABA_CORE_MATRISOME |
| NABA MATRISOME ASSOCIATED | https://www.gsea-msigdb.org/gsea/msigdb/cards/NABA_MATRISOME_ASSOCIATED |
| NABA BASEMENT MEMBRANES | https://www.gsea-msigdb.org/gsea/msigdb/cards/NABA_BASEMENT_MEMBRANES |
| NABA MATRISOME | https://www.gsea-msigdb.org/gsea/msigdb/cards/NABA_MATRISOME |
| REACTOME DEGRADATION OF THE EXTRACELLULAR MATRIX | https://www.gsea-msigdb.org/gsea/msigdb/cards/REACTOME_DEGRADATION_OF_THE_EXTRACELLULAR_MATRIX |
| REACTOME CELL EXTRACELLULAR MATRIX INTERACTIONS | https://www.gsea-msigdb.org/gsea/msigdb/cards/REACTOME_CELL_EXTRACELLULAR_MATRIX_INTERACTIONS |
| GO REGULATION OF RESPONSE TO EXTRACELLULAR STIMULUS | http://www.gsea-msigdb.org/gsea/msigdb/cards/GOBP_REGULATION_OF_RESPONSE_TO_EXTRACELLULAR_STIMULUS.html |
| GO CELLULAR RESPONSE TO EXTRACELLULAR STIMULUS | http://www.gsea-msigdb.org/gsea/msigdb/geneset_page.jsp?geneSetName=GOBP_CELLULAR_RESPONSE_TO_EXTRACELLULAR_STIMULUS |
| GO EXTRACELLULAR MATRIX ASSEMBLY | http://www.gsea-msigdb.org/gsea/msigdb/cards/GOBP_REGULATION_OF_EXTRACELLULAR_MATRIX_ASSEMBLY |
| GO REGULATION OF EXTRACELLULAR MATRIX ORGANIZATION | http://www.gsea-msigdb.org/gsea/msigdb/cards/GOBP_REGULATION_OF_EXTRACELLULAR_MATRIX_ORGANIZATION |
| GO REGULATION OF EXTRACELLULAR MATRIX ASSEMBLY | http://www.gsea-msigdb.org/gsea/msigdb/cards/GOBP_REGULATION_OF_EXTRACELLULAR_MATRIX_ASSEMBLY |
| GO POSITIVE REGULATION OF EXTRACELLULAR MATRIX ORGANIZATION | http://www.gsea-msigdb.org/gsea/msigdb/cards/GOBP_POSITIVE_REGULATION_OF_EXTRACELLULAR_MATRIX_ORGANIZATION |
| GO NEGATIVE REGULATION OF RESPONSE TO EXTRACELLULAR STIMULUS | http://www.gsea-msigdb.org/gsea/msigdb/cards/GOBP_NEGATIVE_REGULATION_OF_RESPONSE_TO_EXTERNAL_STIMULUS |
| GO EXTRACELLULAR REGULATION OF SIGNAL TRANSDUCTION | http://www.gsea-msigdb.org/gsea/msigdb/cards/GOBP_EXTRACELLULAR_REGULATION_OF_SIGNAL_TRANSDUCTION |
| GO RESPONSE TO EXTRACELLULAR STIMULUS | http://www.gsea-msigdb.org/gsea/msigdb/cards/GOBP_RESPONSE_TO_EXTRACELLULAR_STIMULUS |
| GO POSITIVE REGULATION OF RESPONSE TO EXTRACELLULAR STIMULUS | http://www.gsea-msigdb.org/gsea/msigdb/cards/GOBP_RESPONSE_TO_EXTRACELLULAR_STIMULUS |
| GO EXTRACELLULAR MATRIX DISASSEMBLY | https://www.ebi.ac.uk/QuickGO/term/GO:0022617 |
| GO REGULATION OF EXTRACELLULAR MATRIX DISASSEMBLY | http://www.gsea-msigdb.org/gsea/msigdb/cards/GOBP_REGULATION_OF_EXTRACELLULAR_MATRIX_DISASSEMBLY |
| GO EXTRACELLULAR STRUCTURE ORGANIZATION | https://www.gsea-msigdb.org/gsea/msigdb/cards/GO_EXTRACELLULAR_STRUCTURE_ORGANIZATION |
| GO EXTRACELLULAR MATRIX | https://www.gsea-msigdb.org/gsea/msigdb/cards/GO_EXTRACELLULAR_MATRIX |
| GO EXTRACELLULAR MATRIX COMPONENT | http://www.gsea-msigdb.org/gsea/msigdb/geneset_page.jsp?geneSetName=GOMF_EXTRACELLULAR_MATRIX_STRUCTURAL_CONSTITUENT |
| GO EXTRACELLULAR MATRIX STRUCTURAL CONSTITUENT | https://www.ebi.ac.uk/QuickGO/term/GO:0005201 |
| GO EXTRACELLULAR MATRIX BINDING | https://www.ebi.ac.uk/QuickGO/term/GO:0050840 |
| GO EXCITATORY EXTRACELLULAR LIGAND GATED ION CHANNEL ACTIVITY | https://www.ebi.ac.uk/QuickGO/term/GO:0005231 |
| GO EXTRACELLULAR LIGAND GATED ION CHANNEL ACTIVITY | https://www.ebi.ac.uk/QuickGO/term/GO:0005230 |
| GO REGULATION OF EXTRACELLULAR MATRIX CONSTITUENT SECRETION | http://www.gsea-msigdb.org/gsea/msigdb/geneset_page.jsp?geneSetName=GOBP_REGULATION_OF_EXTRACELLULAR_MATRIX_CONSTITUENT_SECRETION |
| GO NEGATIVE REGULATION OF EXTRACELLULAR MATRIX DISASSEMBLY | https://www.ebi.ac.uk/QuickGO/term/GO:0010715 |
| GO SEQUESTERING OF EXTRACELLULAR LIGAND FROM RECEPTOR | http://www.gsea-msigdb.org/gsea/msigdb/geneset_page.jsp?geneSetName=GOBP_SEQUESTERING_OF_EXTRACELLULAR_LIGAND_FROM_RECEPTOR |
| GO EXTRACELLULAR MATRIX CONSTITUENT SECRETION | https://www.ebi.ac.uk/QuickGO/term/GO:0070278 |
| GO PROTEIN LOCALIZATION TO EXTRACELLULAR REGION | https://www.ebi.ac.uk/QuickGO/term/GO:0071692 |
| GO EXTRACELLULAR EXOSOME ASSEMBLY | https://www.ebi.ac.uk/QuickGO/term/GO:0071971 |
| GO POSITIVE REGULATION OF EXTRACELLULAR MATRIX DISASSEMBLY | https://www.ebi.ac.uk/QuickGO/term/GO:0090091 |
| GO EXTRACELLULAR VESICLE BIOGENESIS | https://www.ebi.ac.uk/QuickGO/term/GO:0140112 |
| GO POSITIVE REGULATION OF EXTRACELLULAR MATRIX ASSEMBLY | https://www.ebi.ac.uk/QuickGO/term/GO:1901203 |
| GO NEGATIVE REGULATION OF EXTRACELLULAR MATRIX ORGANIZATION | https://www.ebi.ac.uk/QuickGO/term/GO:1903054 |
| GO COLLAGEN CONTAINING EXTRACELLULAR MATRIX | https://www.ebi.ac.uk/QuickGO/term/GO:0062023 |
| GO EXTRACELLULAR MEMBRANE BOUNDED ORGANELLE | https://www.ebi.ac.uk/QuickGO/term/GO:0065010 |
| GO EXTRACELLULAR MATRIX STRUCTURAL CONSTITUENT CONFERRING TENSILE STRENGTH | https://www.gsea-msigdb.org/gsea/msigdb/cards/GOMF_EXTRACELLULAR_MATRIX_STRUCTURAL_CONSTITUENT_CONFERRING_TENSILE_STRENGTH.html |
| GO EXTRACELLULAR MATRIX STRUCTURAL CONSTITUENT CONFERRING COMPRESSION RESISTANCE | https://www.gsea-msigdb.org/gsea/msigdb/cards/GOMF_EXTRACELLULAR_MATRIX_STRUCTURAL_CONSTITUENT_CONFERRING_COMPRESSION_RESISTANCE.html |
| GO EXTRACELLULAR MATRIX CONSTITUENT CONFERRING ELASTICITY | https://www.gsea-msigdb.org/gsea/msigdb/cards/GOMF_EXTRACELLULAR_MATRIX_CONSTITUENT_CONFERRING_ELASTICITY.html |
| GO EXTRACELLULAR MATRIX PROTEIN BINDING | http://www.gsea-msigdb.org/gsea/msigdb/cards/GOMF_EXTRACELLULAR_MATRIX_PROTEIN_BINDING |

**Supplementary table 2.** A total of 47 ECM gene sets containing 1936 unique ECM-related genes were obtained.

| rank | cmap name | mean | n | enrichment | p-value | specificity | percent non-null |
| --- | --- | --- | --- | --- | --- | --- | --- |
| 1 | verteporfin | 0.893 | 3 | 0.988 | 0.00002 | 0 | 100 |
| 2 | molindone | 0.717 | 4 | 0.89 | 0.00014 | 0 | 100 |
| 3 | **podophyllotoxin** | -0.711 | 4 | -0.905 | 0.00016 | 0.0196 | 100 |
| 4 | sotalol | 0.623 | 4 | 0.883 | 0.00024 | 0 | 100 |
| 5 | mebhydrolin | -0.54 | 4 | -0.893 | 0.00026 | 0 | 100 |
| 6 | chlorhexidine | -0.502 | 5 | -0.832 | 0.00032 | 0 | 100 |
| 7 | Prestwick-1080 | 0.741 | 4 | 0.875 | 0.00034 | 0 | 100 |
| 8 | **lasalocid** | -0.641 | 4 | -0.857 | 0.00074 | 0.0185 | 100 |
| 9 | calcium folinate | -0.308 | 5 | -0.786 | 0.00084 | 0.0153 | 60 |
| 10 | diphenhydramine | -0.534 | 5 | -0.783 | 0.00088 | 0 | 80 |
| 11 | **MG-262** | -0.598 | 3 | -0.918 | 0.00094 | 0.063 | 100 |
| 12 | **nystatin** | -0.597 | 3 | -0.912 | 0.00122 | 0 | 100 |
| 13 | monensin | -0.503 | 6 | -0.708 | 0.00153 | 0.065 | 83 |
| 14 | cefapirin | 0.543 | 4 | 0.821 | 0.00181 | 0 | 100 |
| 15 | lisuride | -0.342 | 5 | -0.742 | 0.00222 | 0.0656 | 60 |
| 16 | todralazine | 0.612 | 5 | 0.752 | 0.00228 | 0.0081 | 100 |
| 17 | levcycloserine | 0.589 | 4 | 0.813 | 0.00237 | 0.0085 | 100 |
| 18 | alprostadil | -0.38 | 7 | -0.64 | 0.00242 | 0.016 | 71 |
| 19 | nalbuphine | 0.541 | 5 | 0.745 | 0.00248 | 0 | 100 |
| 20 | PF-01378883-00 | -0.428 | 4 | -0.805 | 0.00282 | 0 | 100 |

**Supplementary table 3.** Result of CMap analysis.
