# Supplementary figures and images for: A Novel Extracellular Matrix Gene-Based Prognostic Model to Predict Overall Survive in Patients With Glioblastoma
Source: Front Genet. 2022 Jun 17;13:851427. doi: 10.3389/fgene.2022.851427 (PMC9247148; doi:10.3389/fgene.2022.851427)

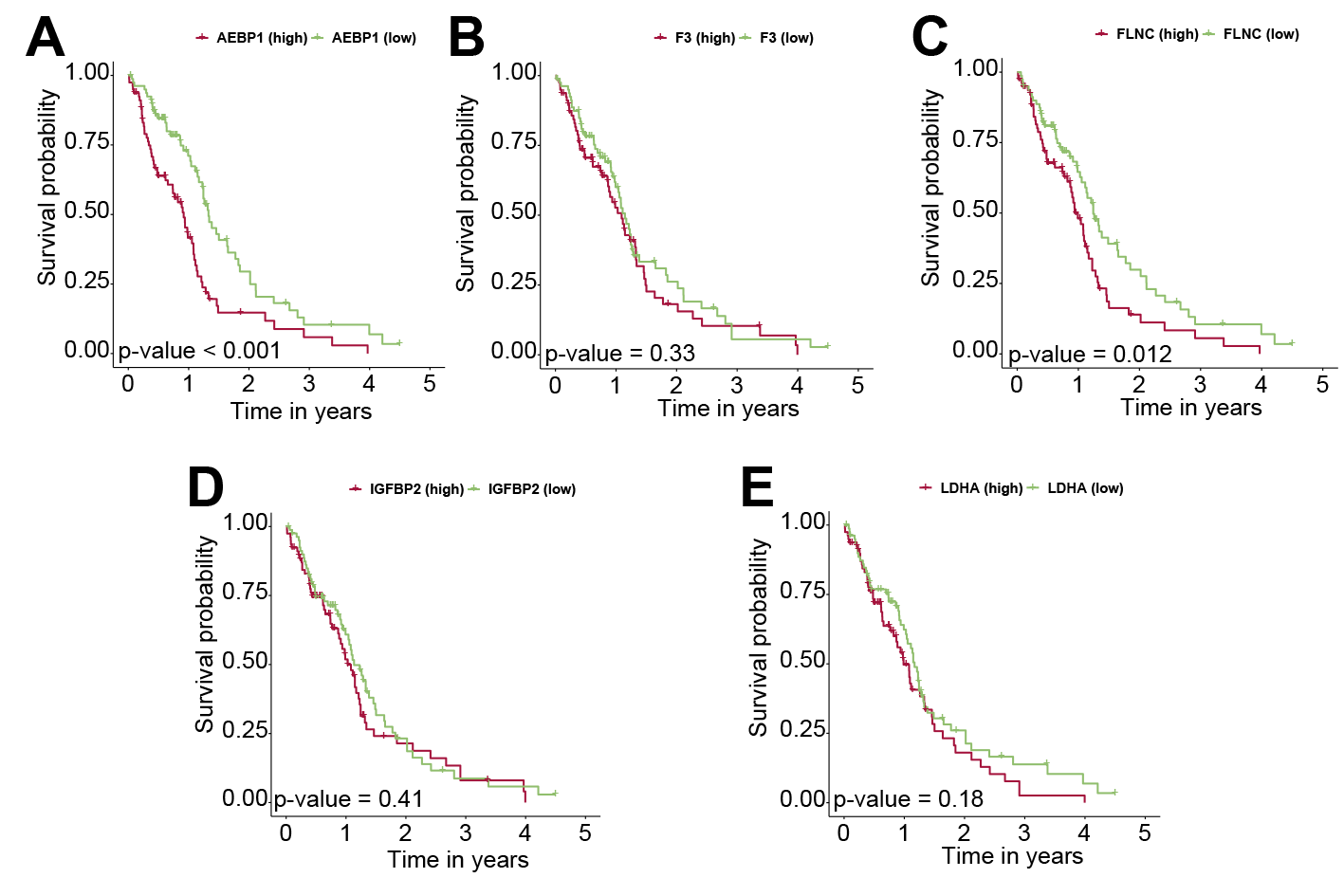

Supplement: Supplementary file 2 [file Image3.TIF]

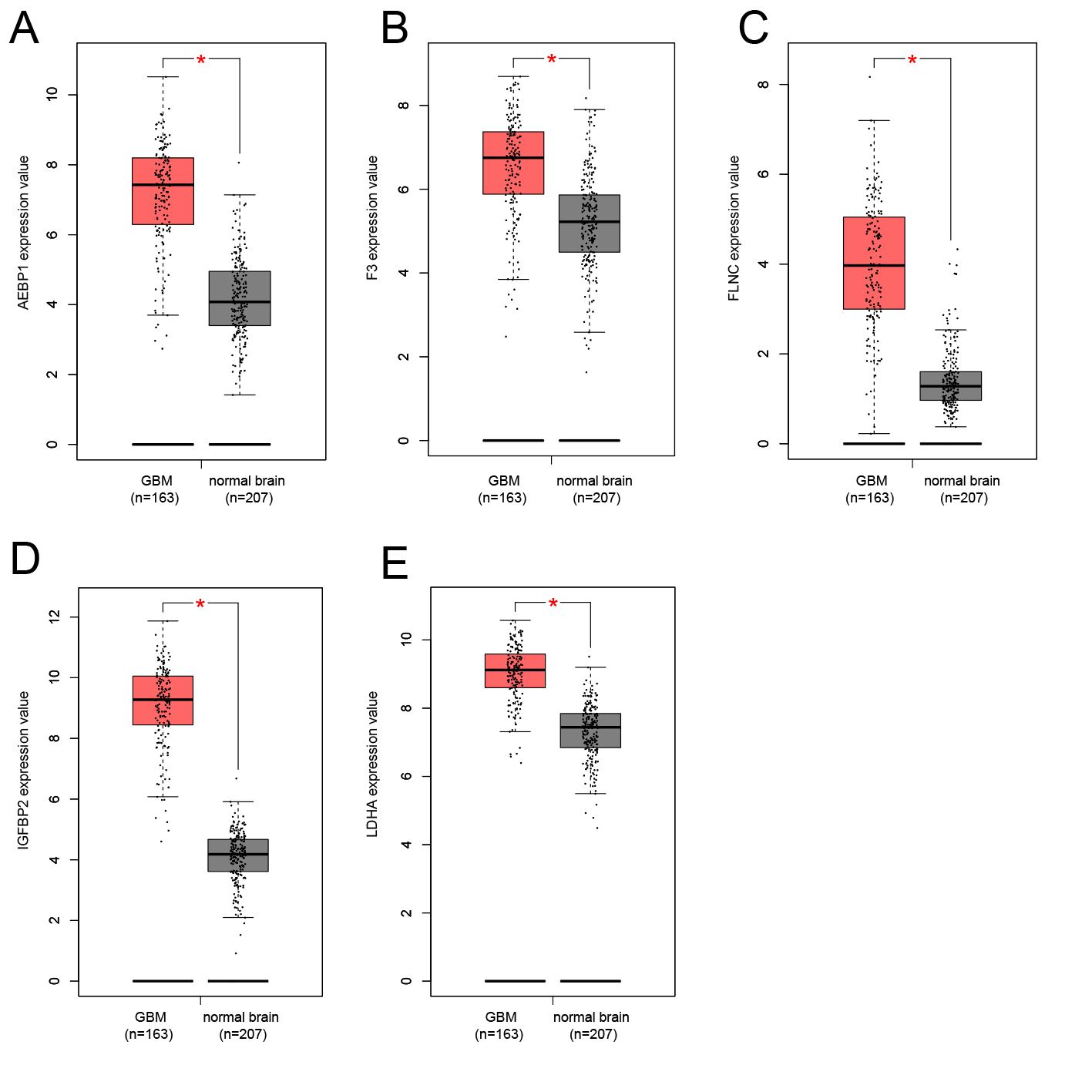

Supplement: Supplementary file 3 [file Image4.TIF]

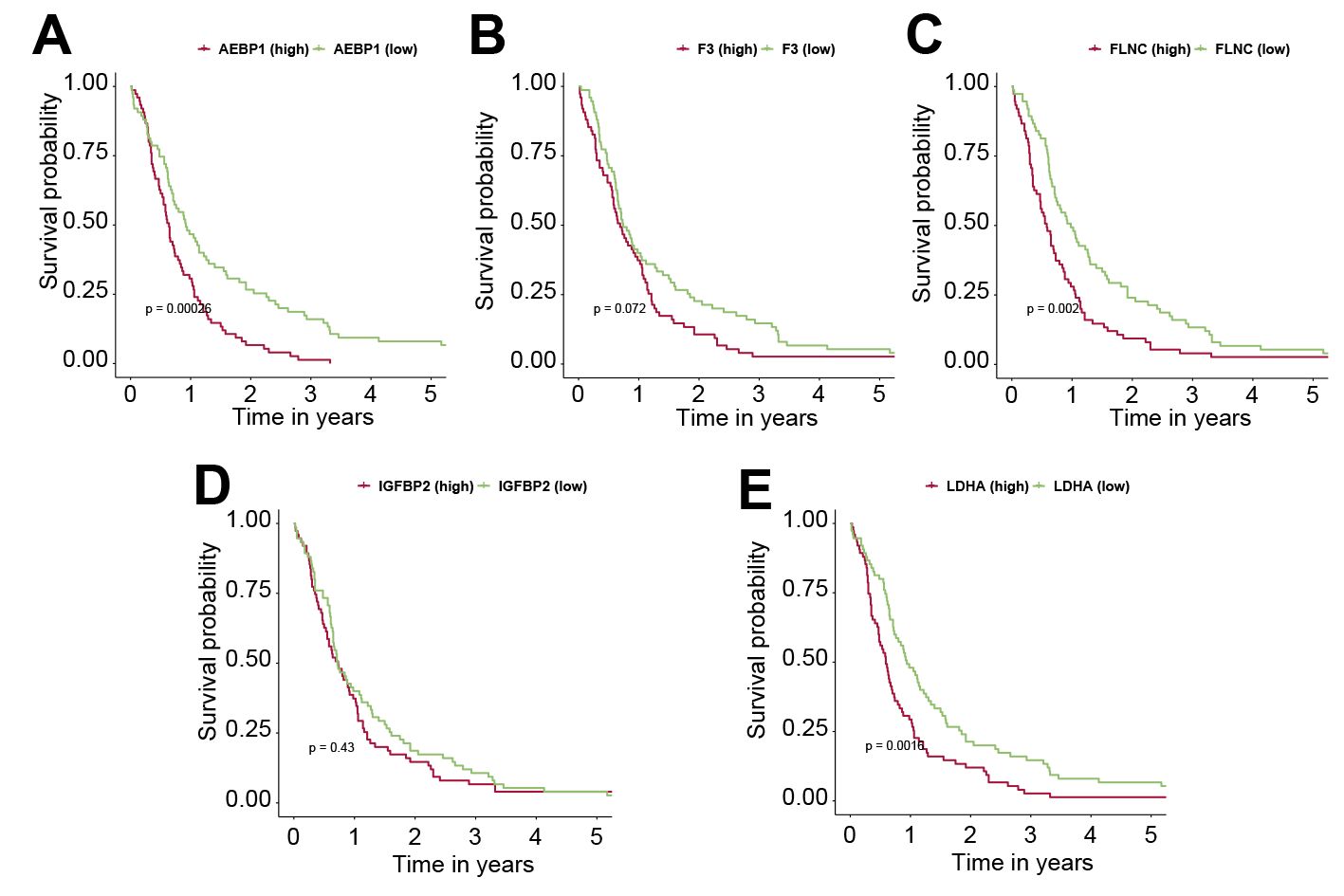

Supplement: Supplementary file 4 [file Image2.TIF]

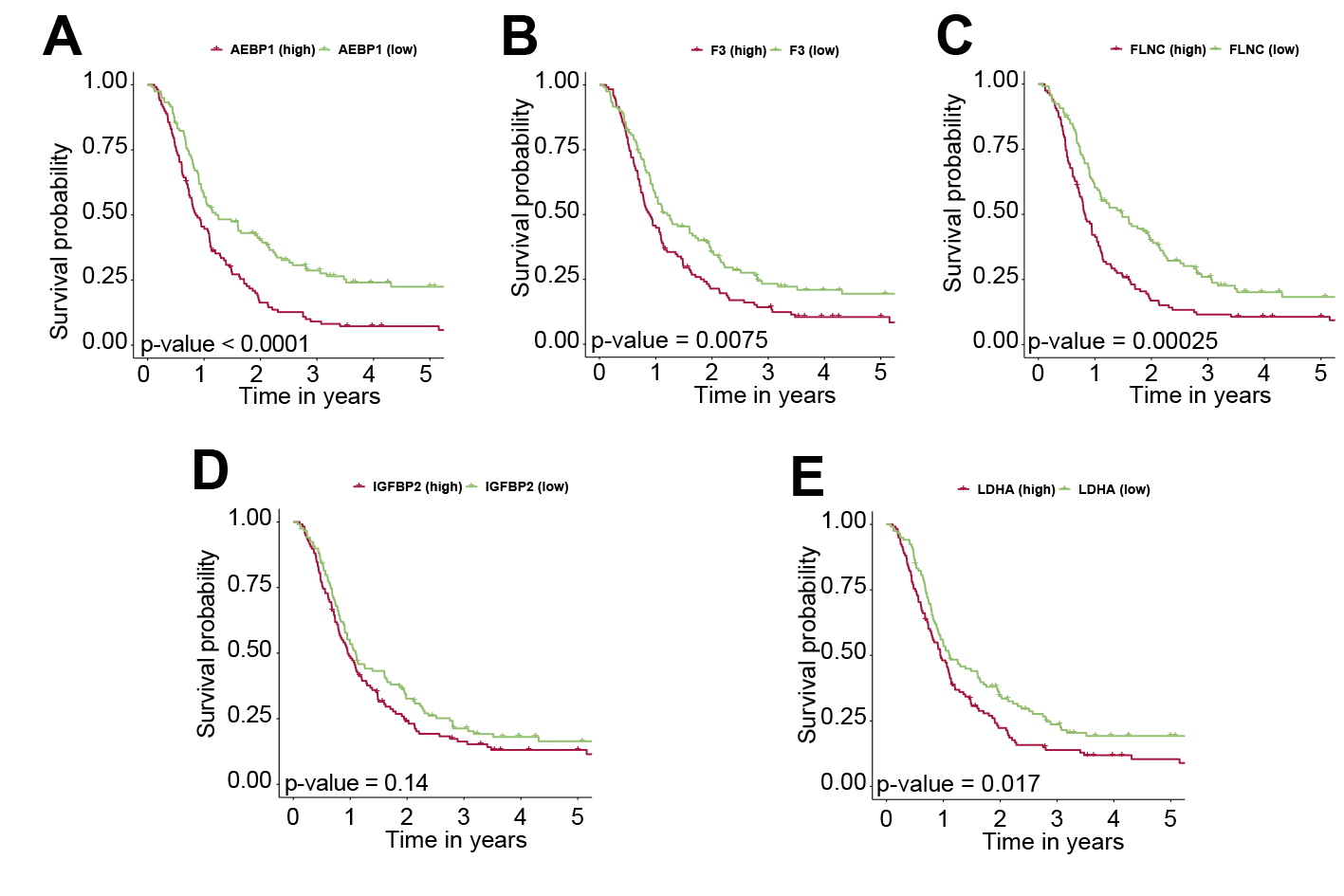

Supplement: Supplementary file 5 [file Image1.TIF]

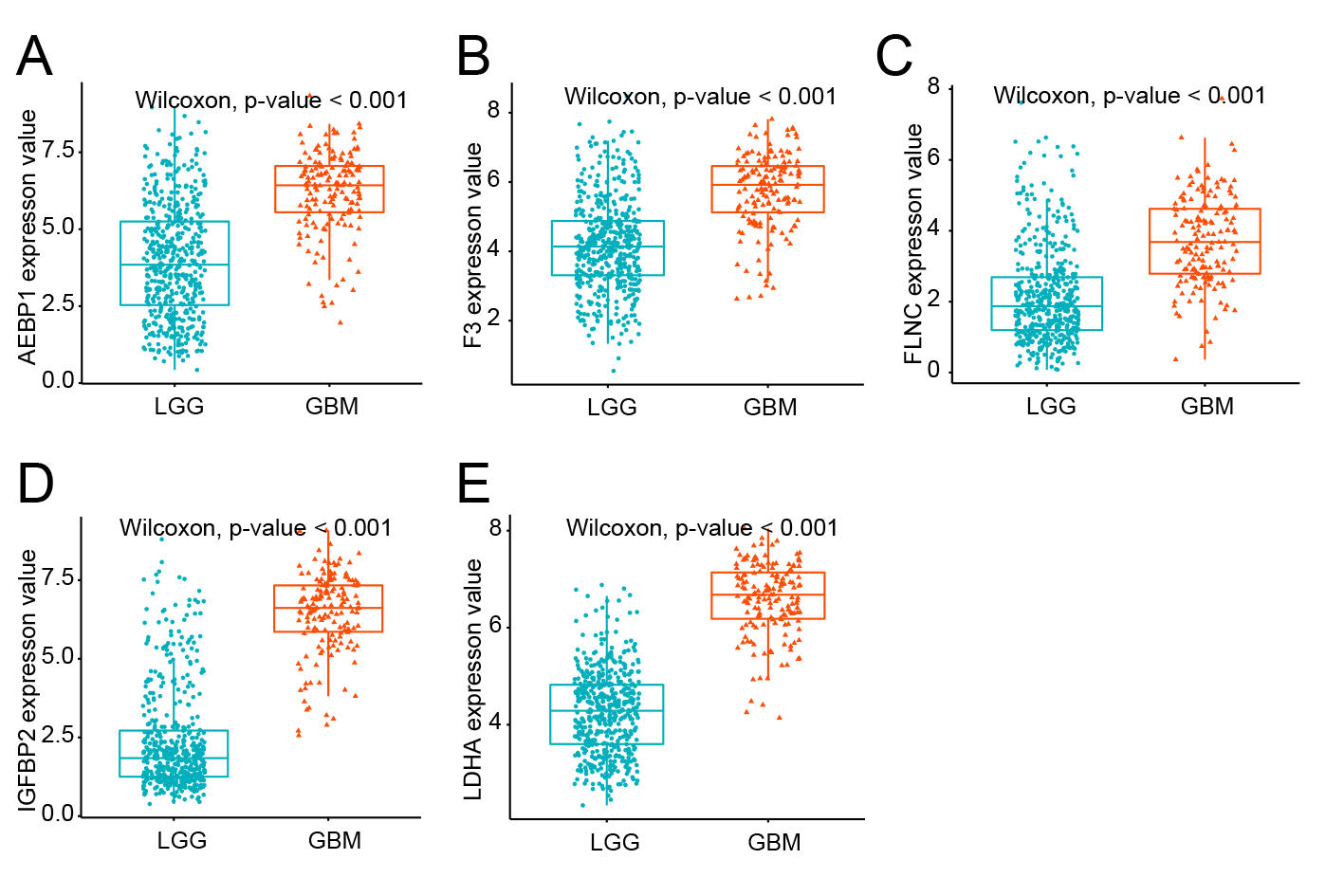

Supplement: Supplementary file 6 [file Image5.TIF]
